# Anti-Mullerian Hormone-to-Testosterone Ratio is Predictive of Positive Sperm Retrieval in Men with Idiopathic Non-Obstructive Azoospermia

**DOI:** 10.1038/s41598-017-17420-z

**Published:** 2017-12-15

**Authors:** Massimo Alfano, Eugenio Ventimiglia, Irene Locatelli, Paolo Capogrosso, Walter Cazzaniga, Filippo Pederzoli, Nicola Frego, Rayan Matloob, Antonino Saccà, Luca Pagliardini, Paola Viganò, Pietro Zerbi, Manuela Nebuloni, Marina Pontillo, Francesco Montorsi, Andrea Salonia

**Affiliations:** 1 Division of Experimental Oncology/Unit of Urology, URI, IRCCS Ospedale San Raffaele, Milan, Italy; 2 Università Vita-Salute San Raffaele, Milan, Italy; 3 Department of Urology, AO Papa Giovanni XXIII, Bergamo, Italy; 4 Infertility Unit, Unit of Obstetrics/Gynecology, IRCCS Ospedale San Raffaele, Milan, Italy; 5 Pathology Unit, Department of Biomedical and Clinical Sciences, L. Sacco Hospital, Università degli Studi di Milano, Milan, Italy; 6 Laboratory Medicine Service, IRCCS Ospedale San Raffaele, Milan, Italy

## Abstract

The lack of clinically-reliable biomarkers makes impossible to predict sperm retrieval outcomes at testicular sperm extraction (TESE) in men with non-obstructive azoospermia (NOA), resulting in up to 50% of unnecessary surgical interventions. Clinical data, hormonal profile and histological classification of testis parenchyma from 47 white-Caucasian idiopathic NOA (iNOA) men submitted to microdissection TESE (microTESE) were analyzed. Logistic regression analyses tested potential clinical predictors of positive sperm retrieval. The predictive accuracy of all variables was evaluated using the receiver operating characteristic-derived area under the curve, and the clinical net benefit estimated by a decision-curve analysis (DCA). Overall, 23 (49%) and 24 (51%) patients were classified as positive and negative sperm retrievals at microTESE. While circulating hormones associated to a condition of primary hypogonadism did not predict sperm retrieval, levels of anti-Mullerian hormone (AMH) and the ratio AMH-to-total Testosterone (AMH/tT) achieved independent predictor status for sperm retrieval at microTESE, with a predictive accuracy of 93% and 95%. Using cutoff values of <4.62 ng/ml for AMH and <1.02 for AMH/tT, positive sperm retrieval was predicted in all individuals, with 19 men out of 47 potentially spared from surgery. DCA findings demonstrated clinical net benefit using AMH and AMH/tT for patient selection at microTESE.

## Introduction

The relevance of male infertility has progressively grown in Western societies, with significant medical, psychological, and socio-economic implications. Seven out of 100 men are infertile, with up to 40% of infertile conditions still of unexplained or idiopathic origin^1–3^. Azoospermia affects about 1% among all men and 10–15% of infertile men^4,5^. With no sperm found at multiple semen analyses, non-obstructive azoospermia (NOA) is the most severe form of infertility^1,5^. Despite genetic causes have been associated with male infertility^3,6,7^, genetic defects are found only in 17–20% of NOA individuals^7–10^. Except for a portion of patients with central endocrine disorders, the remaining 80% of NOA men having negative results on genetic testing are classified as idiopathic NOA (iNOA)^11^.

In patients with clinical evidence of NOA, testicular sperm extraction (TESE) is the technique of choice, which rationally has to be planned as a part of *in vitro* fertilization programs; in this context, microdissection TESE (microTESE) has been advocated as the gold-standard technique to improve sperm yield with minimal tissue excision^12^. However, the lack of useful predictive biomarkers suggestive for successful sperm retrieval at microTESE in NOA men still represents a relevant gap with a very negative return for the patient. Indeed, no significant association has been found between microTESE sperm retrieval outcomes and (i) preoperative testicular volume, (ii) baseline follicular stimulating hormone (FSH) levels, (iii) basal level of Testosterone (T) or increased T level following treatments with aromatase inhibitors, clomiphene citrate or human chorionic gonadotropin^13,14^. Thereof, we sought to identify novel and user-friendly prognostic factors reliably predicting surgical outcomes in iNOA men in the real-life setting. Among other variables, we considered serum levels of testis-derived hormones than might be representative of the primary testicular failure, such as the Anti-Müllerian Hormone (AMH), which is suggestive for a Sertoli cells’ immature phenotype^15^, and T. Of biological relevance, over the adult life AMH expression is under the control of FSH and the inhibitory action of T^16^. Likewise, a paracrine effect of AMH was reported on Leydig cells, with an inhibition of steroidogenesis^17^. For the specific purpose of this study, we considered the ratio of AMH-to-total T (AMH/tT) as a potential effective biomarker to predict the severity of the primary failure of the testis parenchyma.

## Materials and Methods

### Study population

Complete data (clinical characteristics; hormonal profile; sperm retrieval outcomes at surgery; histology; and, reproductive outcomes) from the last 47 white-Caucasian men with iNOA submitted to microTESE at two tertiary-referral centers (Ospedale San Raffaele-Milan-Italy, and Azienda Ospedaliera Papa Giovanni XXIII-Bergamo-Italy) were analyzed in a retrospective study.

According to the World Health Organisation (WHO) criteria, infertility was defined as not conceiving a pregnancy after at least 12 months of unprotected intercourse regardless of whether or not a pregnancy ultimately occurred^18^. Primary infertility was defined as when a couple had never been able to conceive^18^. Men with iNOA were included in the study when having no spermatozoa because of non-obstructive causes in at least two consecutive semen analyses according to the WHO criteria^5^. Idiopathic NOA was defined after comprehensive diagnostic evaluations of all know causes for non-obstructive azoospermia. Thereof, patients with the following clinical features were excluded from the study: azoospermic patients with (i) testicular factors previously associated with infertility (cryptorchidism; grade II and III varicocele; disturbance of erection/ejaculation); (ii) genetic abnormalities previously associated to azoospermia, thus considering mutations of the cystic fibrosis conductance regulator gene (CFTR) associated with congenital bilateral absence of the vas deferens such as CFTR F508del, CFTR F508del heterozygosis, CFTR 5 T/7 T, CFTR 7 T/7 T, and CFTR poly 7 T/9 T, homo and heterozygosis 1298 A > C for the MTHFR gene; microdeletions on the Y chrosomome such as AZFa/b/c; Klinefelter or Kallman syndromes; (iii) known hypothalamic/pituitary defects; (iv) either pituitary or testicular surgery and/or previous vasectomy; (v) previous tumors, including testicular tumors; (vi) testosterone replacement therapy; and, (vii) any other known reason for genital tract obstruction. Conversely, inclusion criteria were (i) a clinical diagnosis of iNOA associated with primary couple’s infertility; (ii) age ≤ 45 years; (iii) white-Caucasian race; (iv) freedom from any known viral and bacterial infections and antibiotic therapies at the time of surgery and throughout the preceding 6 months; and, (v) a comprehensive blood set of analyses over the 12 months before surgery. Weight and height were measured for each participant, and body mass index (BMI), defined as weight in kilograms by height in square meters, was calculated. Waist circumference was measured for every patient^19^. Testes volume was assessed through a Prader orchidometer, calculating the mean value between the two sides.

Patients were then dichotomized according to the surgical outcome (namely, positive versus negative sperm retrieval at microTESE).

### MicroTESE, histology and sperm retrieval

All men underwent microTESE according to the original surgical technique^20^ at two high volume centers for Reproductive Medicine. At the time of testicular microdissection, the parenchyma was immediately placed in 5 ml of Quinn’s™ Sperm Washing Medium (Origio, Måløv, Norway) and minced mechanically with sterile slides. The sample was then transferred into a Falcon tube and centrifuged at 600 g for 10 minutes. The pellet was suspended in a minimum volume of 0.5 ml Quinn’s™ Sperm Washing Medium. Sperm retrieval was checked under an inverted microscope at ×400 magnifications. Sperm were counted, and sperm retrieval was expressed as the number of sperm/high power field (HPF) and then eventually cryopreserved. For the freezing procedure, an equal volume of Quinns Advantage™ Sperm Freezing Medium (Origio, Måløv, Norway) was added to the suspended sperm pellet, and then homogenized and placed at room temperature for 20 minutes. After the room temperature incubation, the sample was heat sealed in a variable number of paillettes CBS™ (Cryo Bio System, L’Aigle, France) and placed at 4 °C for 5 minutes. As a final step, all the paillettes were placed in liquid nitrogen vapor for 15 minutes and then stored in liquid nitrogen. For thawing, the paillettes were warmed to 37 °C and then opened to transfer the sample into a 10 ml Falcon tube. Sample was then washed again with Quinn’s™ Sperm Washing Medium and centrifuged at 600 g for 10 minutes. The supernatant was discarded and samples suspended in variable amounts medium.

A formal histological examination of the testicular specimen was also performed on Bouin and formalin-fixed specimens, followed by hematoxylin-eosin staining. Records were then discussed according to the reported indications^21^. Histological classification of human spermatogenesis was carried out according to the criteria suggested by either Johnsen^22^ or McLachlan^21^. Morphological evaluation of Leydig cell compartment was also performed, and reported as (i) normal Leydig cells, (ii) Leydig celle hyperplasia (multiple nodules, <0.5 cm in diameter, of proliferative Leydig cells) and (iii) Leydig cell tumour (isolate, <3 cm in diameter, well delimitated solid nodule of proliferative atypical Leydig cells).

### Hormonal profile

Venous blood samples were drawn from each patient (7–11 AM) after an overnight fast. Follicle-stimulating hormone (FSH; range of linearity = 0.3–200 mUI/ml, coefficient of variability = 1.9%), luteinizing hormone (LH; range of linearity = 0.3–200 mUI/ml, coefficient of variability = 2.9%), thyroid-stimulating hormone (TSH; range of linearity = 0.005–100 µUI/ml, coefficient of variability = 4.7%), and 17β-estradiol (E_2_; range of linearity = 5–3000 pg/ml, coefficient of variability = 3.9%) were measured using a heterogeneous competitive magnetic separation assay (Bayer Immuno 1 System; Bayer Corporation). An enzyme-linked immunosorbent assay was used to measure Inhibin B (Inhibin B Gen II ELISA, Beckman Coulter; range of linearity = 2.6–1000 pg/ml, coefficient of variability = 4.3%) and AMH (AMH Gen II ELISA; Beckman Coulter; range of linearity = 0.01–23 ng/ml, coefficient of variability = 3.6%). Electrochemiluminescence immunoassay was used for measuring total T levels (Elecsys Testosterone II, Roche; range of linearity = 0.025–15 ng/ml, coefficient of variability = 3.2%) and Prolactin (Elecsys Prolactin II, Roche; range of linearity = 0.1–470 ng/ml, coefficient of variability = 4.3%). Sex hormone–binding globulin (SHBG) levels were measured via a solid-phase chemiluminescent immunometric assay on the Immulite 2000 (Medical Systems SpA; range of linearity = 0.02–180 nmol/L, coefficient of variability = 3.8%). The same laboratory was used for all patients.

### Statistical methods

Continuous variables are presented as medians and interquartile ranges (IQR). Mann-Whitney test was used for comparing continuous variables, and Chi^2^ test for categorical data.

The statistical analyses consisted of several steps. First, logistic regression model estimated odds ratios (OR) and 95% confidence intervals (CIs) for the univariable (UVA) and multivariable (MVA) association between AMH, AMH/tT and a positive sperm retrieval at microTESE, after adjusting for covariates previously reported to be associated with primary testicular failure in NOA men (e.g., age, FSH levels, and mean testicular volume). The Receiver Operating Characteristic (ROC) curve was obtained to quantify the predictive accuracy (Area Under the Curve, AUC) of AMH and the AMH/tT^23^. Then, AMH and AMH/tT values were dichotomized according to the most informative cut-off value capable of maximizing sensitivity when predicting positive sperm retrieval. Finally, we used decision-curve analysis (DCA) to evaluate the clinical net-benefit of the two predictive markers^24^. Statistical analyses were performed using GraphPad Prism 5.9; DCA was computed using R version 3.3.0 (2016, The R Foundation for Statistical Computing, www.r-project.org). All tests were two-sided, with a significance level set at 0.05.

### Study approval

Data collection followed the principles outlined in the Declaration of Helsinki; all patients signed an informed consent agreeing to provide their own anonymous information and tissue specimens for future studies. The study was approved by the Institutional Review Board (Authorization Protocol URI001-2010, further amended on December 2015 by the Ethic Committee IRCCS Ospedale San Raffaele, Milan, Italy).

## Results

Table 1 depicts descriptive statistics of the entire cohort of patients, further stratified according to sperm retrieval outcomes at microTESE. Overall, 23 (49%) and 24 (51%) patients were classified as positive and negative sperm retrievals, respectively. Of the 23 men with a positive sperm retrieval, 22 (95.6%) underwent monolateral surgery and 1 to bilateral surgery (4.3%); in contrast, all 24 individuals with negative outcomes underwent bilateral microTESE. Groups did not differ in terms of age at surgery, waist circumference, BMI and testicular volume. Likewise, groups were comparable in terms of seminal plasma volume and pH, and circulating levels of FSH, LH, SHBG, TSH, InhB, E_2_ and tT (Table 1). Conversely, median circulating levels of AMH and AMH/tT values were higher in men with negative sperm retrieval (both p < 0.001, Table 1).

**Table 1:** Descriptive statistic of clinical and hormonal profile of men with iNOA (whole cohort; n = 47) according to sperm retrieval outcome at microTESE.

	All iNOA men (n = 47)	Positive sperm retrieval (n = 23)	Negative sperm retrieval (n = 24)	P value	Reference values
	Median (IQR)				
Age at microTESE (Y)	38 (33–40)	39 (32–42)	38 (36–40)	0.6	
Waist circumf. (cm)	94.5 (88.5–99.5)	94.5 (90–98)	95 (88.5–99.5)	0.9	<102
BMI (Kg/m^2^)	25 (23.6–26.6)	25 (23.4–26.6)	25 (23.7–27.4)	0.8	18.5–25
Right testis vol. (ml)	10 (10–12)	11 (10–12)	10 (10–12)	0.9	15–25
Left testis vol. (ml)	10 (8–12)	10 (9–12)	10 (8–12)	0.7	15–25
Seminal volume (ml)	3 (2.5–4)	3 (2–4)	3 (2.7–4)	0.4	1.2–7.6
Seminal plasma pH	8 (7.8–8)	8 (7.8–8)	8 (7.8–8)	0.9	7.2–8
FSH (mUI/ml)	18.3 (12–25.7)	18.3 (12–27)	17.9 (12.3–23.1)	0.6	1.4–18.1
LH (mUI/ml)	7 (4.6–10.6)	7.7 (4.4–11.8)	7 (4.7–10.4)	0.8	1.7–8.6
Prolactin (ng/ml)	8.7 (6.2–13)	7.9 (5.4–10.2)	10 (7.7–15)	0.14	2–18
TSH (µUI/ml)	1.6 (1.3–2.6)	1.6 (1.2–1.2)	2 (1.3–3.1)	0.2	0.25–5
SHBG (nmol/L)	28.5 (21.8–33.9)	29 (21–33.1)	28 (22–35.9)	1	13–71
InhB (pg/ml)	34.8 (7–57.6)	32.7 (7–52)	37.7 (10.3–61.9)	0.7	25–325
E2 (pg/ml)	25 (24–29.4)	25 (24–27)	24 (22.5–31.6)	0.6	<58
tT (ng/ml)	3.7 (3–4.4)	3.9 (2.4–5)	3.6 (3–4.3)	0.5	2.8–8
AMH (ng/ml)	3.43 (2–6)	2.0 (1.09–2.91)	6 (4–9.43)	<0.001	0.77–14.5
AMH/tT	0.91 (0.42–1.84)	0.43 (0.3–0.77)	1.77 (1.13–2.41)	<0.001	

Median and interquartile range (IQR) were calculated from 23 retrieval positive and 24 retrieval negative iNOA men. Statistical significance (P-value) between positive and negative sperm retrieval was evaluated by means of two-tailed Mann Whitney test. Reference value is presented as range (min-max). Testis volume was assessed using a Prader orchidometer. All hormones were evaluated in peripheral blood (serum). All data are from the last examination (0–12 months) preceding microTESE. Y: years; BMI: body mass index; FSH: follicle stimulating hormone; LH: luteinizing hormone; PRL: prolactin; TSH: thyroid stimulating hormone; SHBG: sex hormone binding globulin; InhB: Inhibin B; E2; 17β-estradiol; tT: total testosterone; AMH: Anti-Mullerian hormone.

**Figure 1:**
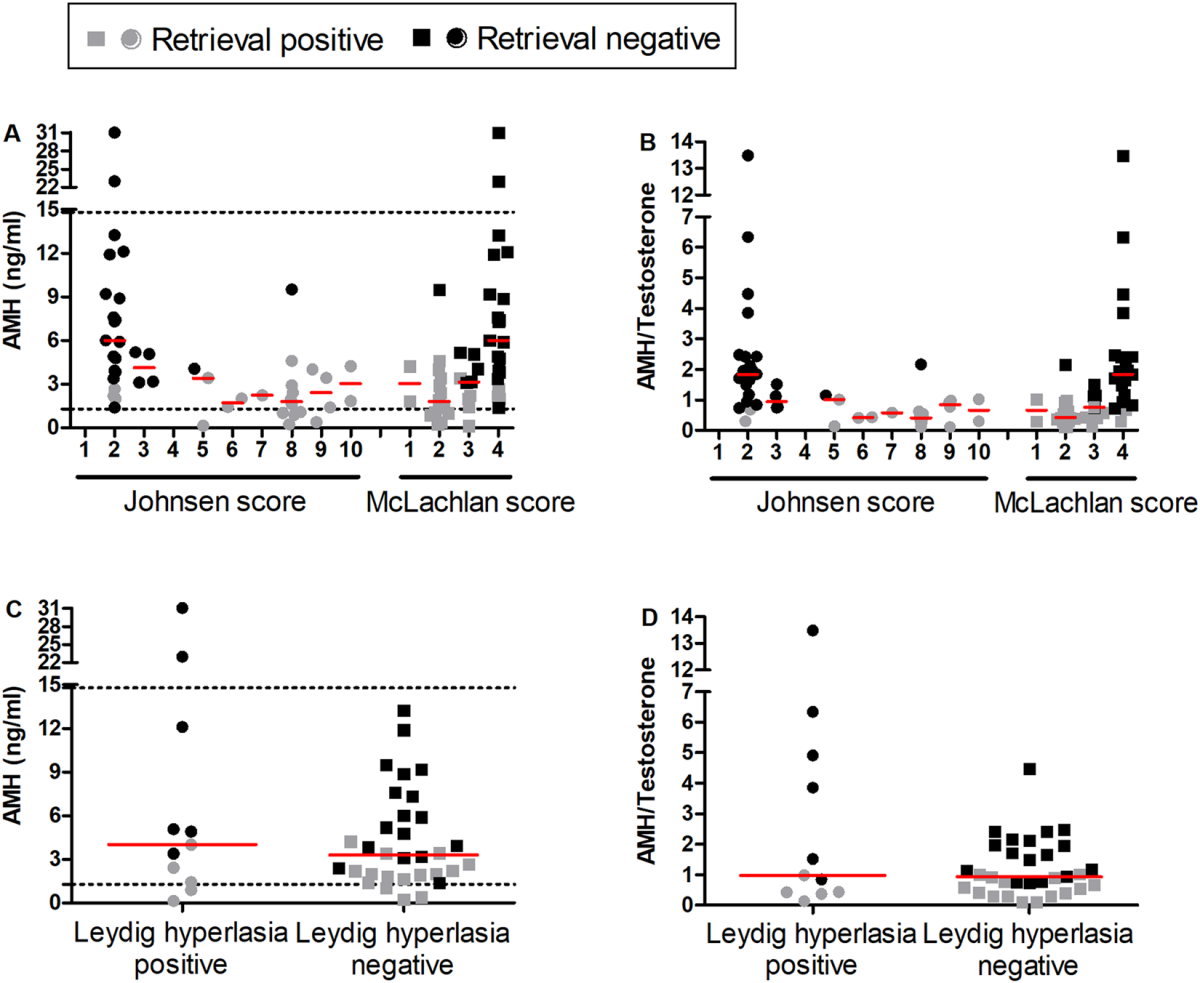
Serum levels of AMH and AMH/tT ratio values did not allow to stratifying histological classification of human spermatogenesis. (**A**,**B**) Descriptive classification of human spermatogenesis was performed using Johnsen’s (43) and McLachlan’s (42) score. Score for assessing spermatogenesis in testicular biopsy according to the Johnsen score: 10 = complete spermatogenesis and perfect tubules; 9 = many spermatozoa present but disorganized spermatogenesis; 8 = few spermatozoa present; 7 = no spermatozoa but many spermatids present; 6 = few spermatids present; 5 = nospermatozoa or spermatids present but many spermatocytes present; 4 = few spermatocytes present; 3 = only spermatogonia present; 2 = no germ cells present; 1 = neither germ cells nor Sertoli cells present. Score for assessing spermatogenesis in testicular biopsy according to the McLachlan score: 1 = normal testicular biopsy, full spermatogenesis in the entire biopsy and the presence of a normal inter-tubular tissue; 2 = hypospermatogenesis, when all stages of spermatogenesis are present but reduced to a varying degree, including varying patterns that can result in some tubules showing an epithelium containing Sertoli cells only; 3 = germ cell arrest, describes the total arrest at a particular stage, most often at the spermatogonial or primary spermatocyte stage; 4 = Sertoli cell only syndrome, when there are no tubules containing germ cells. Dot plots depict AMH levels and AMH/tT ratio values from the 23 positive sperm retrievals and 24 negative sperm retrievals; horizontal bars detail median values. Dashed lines represent the range of reference values for AMH levels. Lack of statistical significance among classes was evaluated by ANOVA. (**C**,**D**) Classification of testis parenchyma based on hyperplasia of Leydig cells. Dot plots show values from the 23 positive sperm retrievals and 24 negative sperm retrievals iNOA men; horizontal bars detail median values. Dashed lines represent the range of reference values for AMH levels. Lack of statistical significance between classes was evaluated by two-tail unpaired T test.

According to both Johnsen’ and McLachlan’ scoring systems of germinal histological classification, testis parenchyma of negative sperm retrieval iNOA men were classified as Sertoli cells only syndrome (SCOS) in 18 out 24 (75%) individuals, complete germ cell arrest in 5 out of 24 (21%), and hypo-spermatogenesis in 1 out of 24 (4%); conversely, the testis parenchyma of positive sperm retrieval patients was classified as SCOS in 3 out of 23 (13%), germ cell arrest in 5 out of 23 (22%), hypospermatogenesis in 13 out of 23 (56.5%) and normal spermatogenesis in 2 out of 23 (8.5%) (Fig. 1A,B). Normal Leydig cells compartment was found in 18 out of 23 (78%) positive sperm retrieval and 18 out of 24 (75%) negative sperm retrieval; Leydig cell hyperplasia was found in 5 out of 23 (22%) positive sperm retrieval and 6 out of 24 (25%) negative sperm retrieval (Fig. 1C,D), whereas no Leydig cell tumour was observed. Serum AMH levels and AMH/tT values were neither significantly associated to any histological classification (Fig. 1A and B) nor to a condition of Leydig cells hyperplasia (Fig. 1C and D). In all conditions, both AMH and AMH/tT values were higher in retrieval negative individuals, despite AMH median value was in the reference range (Fig. 1).

Table 2 details logistic regression analyses for positive sperm retrieval at microTESE. At UVA, AMH levels (OR 0.28, 95% CIs 0.13–0.61, p 0.001) and AMH/tT (OR 0.00, 95% CIs 0–0.13, p 0.004), but not age at surgery, testis volume and FSH levels, emerged as predictors of positive sperm retrieval. Similarly, circulating levels of AMH (MVA Model 1, p = 0.004) and AMH/tT ratio (Model 2, p = 0.008) emerged as independent predictors of positive sperm retrieval, after adjusting for the aforementioned covariates (Table 2).

**Table 2:** Logistic regression analyses for positive sperm retrieval at microTESE.

	Univariable			Multivariable Model 1			Multivariable Model 2		
	OR	95% CI	p	OR	95% CI	p	OR	95% CI	p
Age at microTESE	1.0	0.91–1.12	0.91	0.96	0.8–1.14	0.62	0.94	0.79–1.12	0.5
Right testis volume	0.97	0.83–1.14	0.71	1.13	0.95–1.76	0.11	1.46	0.77–2.78	0.2
FSH	1.03	0.98–1.07	0.31	0.99	0.89–1.09	0.85	1	0.9–1.13	0.87
AMH	0.28	0.13–0.61	0.001	0.21	0.07–0.6	0.004			
AMH/tT	0.00	0.00–0.13	0.004				0.01	0.00–0.17	0.008

OR; odds ratio, CI; confidence intervals. p; statistical significance.

**Figure 2:**
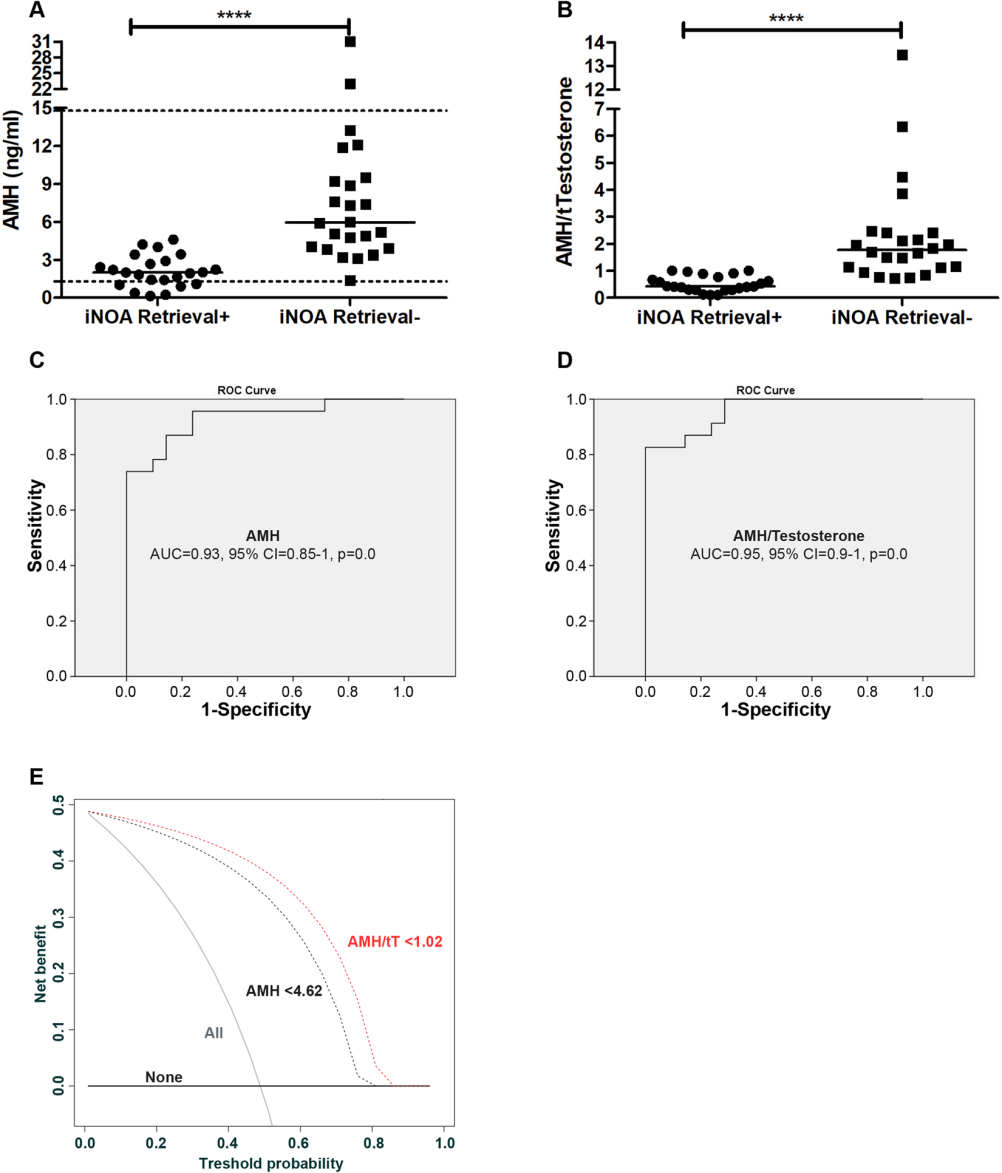
Circulating AMH levels and AMH/tT ratio values were predictive of sperm retrieval in iNOA men undergoing microTESE. (**A**,**B**) Dot plots depict values from the 23 positive sperm retrievals and 24 negative sperm retrievals iNOA men; horizontal bars detail median values. Dashed lines represent the range of reference values for the serum level of AMH. Statistical significance was evaluated by means of two-tail non-parametric T test (Mann-Whitney test). (**C**,**D**) ROC-derived curve, showing the AUC (Area Under the Curve) 95% CI (confidence intervals) and statistical significance. (E) Decision curve analysis showing the net benefit of AMH and AMH/tT on the prediction of positive sperm retrieval in iNOA men undergoing to microTESE. The use of the cutoff of <4.62 ng/ml for AMH and of <1.02 for AMH/tT resulted in positive net benefit; in the absence of any marker the 50% probability of sperm retrieval was associated to a net benefit of 0, whereas the use of the 2 markers at the threshold of 50% provided increased net benefit to 0.3–0.4, with the AMH/tT ratio scoring better than AMH.

To quantify discrimination of the outcome of microTESE based on the blood level of AMH (Fig. 2A) and AMH/tT (Fig. 2B), AUC of the ROC-curve was estimated. The AUC for AMH and AMH/tT were respectively 0.93 (95% CI = 0.85–1, Figs. 2C) and 0.95 (95% CI = 0.9–1, Fig. 2D). Conversely, the predictive performances of testis volume (AUC = 0.49, 95% CI = 0.31–0.60), serum tT (AUC = 0.57, 95% CI = 0.38–0.75), and FSH (AUC = 0.44, 95% CI = 0.26–0.62) were not able to subdivide patients in terms of positive vs. negative sperm retrievals.

The most informative cut-off values for AMH and AMT/tT were then calculated; in this context, circulating AMH levels below 4.62 ng/ml resulted in 100% sensitivity and 82% specificity, with a positive predictive value of 79% and a negative predictive value of 100% for positive sperm retrieval; likewise, AMH/tT values below 1.02 achieved same performances as AMH levels <4.62 ng/ml (Table 3).

**Table 3:** Sensitivity, specificity, positive predictive value and negative predictive value for the cutoff of AMH <4.62 ng/ml and AMH/tT < 1.02.

AMH <4.62 ng/ml; AMH/tT <1.02		Outcome		Total
		Negative at test	Positive at test	
Retrieval Negative	Count	19	5	24
	% within AMH	79.2	20.8	100
	% within outcome	100	17.9	51.1
Retrieval Positive	Count	0	23	23
	% within AMH	0	100	100
	% within outcome	0	82.1	48.9
Total iNOA	Count	19	28	47
	% within AMH	40.4	59.6	100
	% within outcome	100	100	100

Decision-curve analysis showed the net benefit of using both AMH and AMH/tT to identify patients in terms of sperm retrieval outcomes, with a slight advantage in favor of AMH/tT values toward AMH levels (Fig. 2E).

In the testis parenchyma of positive sperm retrievals, a median of 0.038 sperm/high power field (IQR = 0.0033–0.48) were found. At the time of preparation of this manuscript *in vitro* fertilization via intracytoplasmic sperm injection was already attempted in 12 out of 23 couples (52.2%). Of all, 7 embryos were transferred into the uterus, thus resulting in 5 biochemical pregnancies, 5 clinical pregnancies, and 3 newborns.

## Discussion

Still there is a disarming lack of clinically useful predictive biomarkers of sperm retrieval outcomes at testicular surgery in NOA men undergoing *in vitro* fertilization programs. Major achievement of this study is the novel identification of two potential user-friendly biomarkers with high predictive accuracy toward positive sperm retrieval at microTESE. Indeed, we found that circulating AMH levels and AMH/tT values allowed dichotomizing a homogenous cohort of white-Caucasian primary infertile men with iNOA into positive vs. negative sperm retrievals at microdissection testicular surgery, with >93% accuracy. In this context, the adoption of AMH below 4.62 ng/ml or an AMH/tT ratio cutoff value below 1.02 resulted in 100% sensitivity for positive sperm retrieval, at the clinical cost of including 5 out of 28 (18%) false positive but reliably sparing unnecessary microTESE in 19 out of 47 men (40.4%).

Strengths of these findings first come from the confirmation of previous observation that age at surgery, testicular volume and circulating FSH levels were not reliable biomarkers of positive vs. negative sperm retrieval in the real-life scenario. Indeed, even in our relatively small but homogenous cohort of iNOA individuals, age at surgery did not achieve independent predictor status for sperm retrieval outcomes^25^. Similarly, regardless of sperm retrieval outcomes at microTESE, all iNOA men considered in our cohort showed reduced testicular volume. Despite testicular hypotrophy has been advocated to be associated with spermatogenic dysfunction^14^, current findings confirmed previous data showing that testis volume per se is not a reliable predictor of sperm retrieval outcomes at surgery^26^. To this must be added that primary hypogonadism in infertile men has been associated with low tT serum levels and high circulating FSH levels^27,28^. Data from the European Male Aging Study (EMAS) showed that serum levels of tT <3.03 ng/ml and LH >9.4 mU/ml reliably identified primary hypogonadism in aging men^29^. Recently, the same two markers were applied also to stratify different types of hypogonadism in primary infertile men^30^. Current findings confirmed that the aforementioned cutoff values used for FSH, LH and tT did not identify patients with a positive vs. a negative sperm retrieval at surgery, thus confirming known data^31^. More in depth, the circulating levels of FSH, LH and tT did not emerge to adequately define the severity of the primary exocrine testis failure.

Further studies are certainly needed to shed light on the mechanisms behind the condition of germ cell aplasia in idiopathic infertile men. To this aim, the finding of these two novel prognostic biomarkers of sperm retrieval in iNOA men might outline at least two pathogenetic mechanisms. A first interpretation is that while retrieval positive testes emerged to be heterogeneous for the presence of both areas positive for sperm along with seminiferous tubules characterized by germ cell arrest and Sertoli cells only, conversely 75% of sperm retrieval negative testes were classified as SCOS and 21% with complete germ cell arrest. The histological classification of the testis parenchyma was previously associated with the severity of clinical diagnosis in infertile men, but the score was not associated with testicular volume, and serum levels of inhB, FSH, LH and tT^32–34^. In agreement, both serum AMH levels and AMH/tT ratio values were not associated with the histological findings. According to the applied mono vs. bilateral microTESE to find sperms, we may rationally infer that a negative outcome upon bilateral surgery was indicative of the absence of germ cells in both testes; thereof, the increase AMH levels and AMH/tT ratio values were faithful markers of a globally impaired exocrine function of both testes. Hence, being hormonal serum levels representative on the one hand of the production of the entire testis, and on the other of the different degree of the disease, circulating tT and AMH might be considered adequate indicators of the endocrine gonadal system, while an increased AMH/tT ratio in retrieval negative individuals might be representative of a spreading of the pathological insult within the testis, with the potential consequent spreading of SCOS to all seminiferous tubules. A second interpretation is that the gonadal endocrine system of sperm retrieval positive and negative iNOA men may be representative of different stages of testicular development/maturation. The blood level of AMH is high from the neonatal to the pubertal stage in males, with a subsequent decrease to 3–4% of the infant levels throughout the adulthood^35^. In this context, AMH production is under the control of FSH up to the pubertal stage^36^ without being subject to control by androgens^37^; in contrast, AMH level is drastically reduced by T during the adult life^16^. Likewise, a paracrine effect of AMH was reported on Leydig cells, with an inhibition of steroidogenesis^17^. Thereof, it would seem that the main physiological role of AMH in the adult male is limited to the paracrine control of testicular function. To this regard, AMH/tT ratio appeared to be representative of the balance between the exocrine and the endocrine compartments within the testis; therefore, the high blood levels of AMH and AMH/tT ratio observed in negative sperm retrieval iNOA men, with a testicular histology of SCOS along with germ cell aplasia, is likely representative of an impaired control of the androgen toward the expression of AMH. We could speculate that in iNOA individuals with negative sperm retrieval at microTESE, high blood levels of AMH and AMH/tT ratio might be even expression of a sort of testis regression to a pre-puberal stage, with immature Sertoli cells unresponsive to T action.

Blood level of AMH is considered as a predictive marker of ovarian reserve in women^38^, becoming undetectable at menopause^39^; this makes circulating AMH extremely relevant especially in terms of assisted reproductive technology^40^. Conversely, for the first time we showed that elevated blood AMH levels and increased AMH/tT values may reflect the depletion of the germ cell reservoir within the testis in iNOA men. In this context, serum levels of AMH and AMH/tT values appeared as more reliable biomarkers representative of the severity of the primary testis failure.

Blood levels of AMH were previously considered not diagnostically significant in men with either obstructive azoospermia^41^ or oligozoospermia^42^, whereas decreased blood levels of AMH were reported in NOA men^41^. It has been also reported that circulating AMH lacks prognostic significance in terms of sperm retrieval in NOA men submitted to testicular sperm aspiration or TESE^43^. Despite that genetic background^44^ and genetic abnormalities^45^ are known factors modulating the circulating levels of AMH, previously published studies did not report specific inclusion/exclusion criteria regarding the considered infertile population. In this context, we consider a major strength of our study the detailed definition of specific inclusion and exclusion criteria, which at least allow to selecting a homogeneous cohort of idiopathic infertile white-Caucasian men below 45 years of age.

Overall, serum levels of AMH and the AMH/tT ratio hold a number of translational outcomes in clinical terms. Of major clinical relevance, further implementation in the clinical practice would reduce the number of unnecessary surgery, while focusing on patients with the highest chance of sperm retrieval.

Our study is not devoid of limitations. Above all, the study has considered a relatively small cohort of iNOA patients; therefore larger studies across different centers and populations will be needed to substantiate our findings. Second, the strict selection of only white-Caucasian iNOA men might necessarily pave the way for setting novel cut-offs for AMH and AMH/tT in infertile men of different genetic backgrounds.
